# Study on the Selective Inhibition of FeSO_4_-NaH_2_PO_4_ in Sphalerite–Pyrite Flotation Separation

**DOI:** 10.3390/molecules31122137

**Published:** 2026-06-17

**Authors:** Zhiyong Zhang, Jingjing Xiao, Sheng Liu, Pan Xiao, Huijun Cao

**Affiliations:** 1 School of Safety Engineering, Hunan Vocational Institute of Safety Technology, Changsha 410151, China; zzy202308@126.com; 2 State Key Laboratory of Utilization of Woody Oil Resource, Hunan Academy of Forestry, Changsha 410004, China; 19146808384@163.com; 3 College of Chemistry and Chemical Engineering, Central South University, Changsha 410083, China; 4 School of Pharmaceutical and Bioengineering, Hunan Chemical Vocational Technology College, Zhuzhou 412000, China; huijunxiangbin818@163.com

**Keywords:** sphalerite, pyrite, flotation separation, FeSO_4_-NaH_2_PO_4_, selective inhibition and activation

## Abstract

Sphalerite–pyrite separation has always been one of the key issues of concern in the field of mineral flotation. To achieve selective flotation separation of sphalerite and pyrite, a novel flotation system, FeSO_4_+NaH_2_PO_4_-CuSO_4_-SIBX, was adopted. This study validated the novel separation system using mineral flotation experiments. The changes in the hydrophobicity and surface charge of the minerals were evaluated using experiments such as contact angle, zeta potential, and agglomeration. Finally, the mechanism of sphalerite–pyrite separation was revealed through XPS and solution chemistry analysis. The results showed that sphalerite and pyrite were effectively separated by flotation. In the novel flotation system, the synergistic effect of FeSO_4_ and NaH_2_PO_4_ shifted the surface potential of pyrite positively, and subsequent treatment with CuSO_4_ and SIBX did not significantly alter its hydrophobicity. However, sphalerite, after treatment with FeSO_4_+NaH_2_PO_4_-CuSO_4_-SIBX, exhibited significantly enhanced hydrophobicity and marked mineral particle agglomeration. Further research revealed that within the pH range of 6.0–8.0, hydrophilic components formed by Fe^2+^ and H_2_PO_4_^−^ (e.g., Fe(H_2_PO_4_)^+^ and Fe_3_(PO_4_)_2_·8H_2_O) selectively adsorbed onto the pyrite surface, enhancing its hydrophilicity. Meanwhile, sphalerite was activated by the substitution reaction of Cu^2+^ with ZnS, forming a hydrophobic layer on its surface with SIBX in the slurry.

## 1. Introduction

Zinc, as an indispensable key metallic raw material in modern industry, has wide applications in metallurgy, the chemical industry, electronics, energy, and other fields [1,2,3]. Sphalerite (ZnS) is the main raw material for zinc extraction, and its most common associated sulfide gangue mineral, pyrite (FeS_2_), must be effectively separated during processing. Otherwise, it will not only reduce the grade of zinc concentrate but also adversely affect the efficiency and economy of subsequent metallurgical processes. In addition, the sulfur released by unseparated pyrite can lead to environmental hazards such as acid rain [1,4]. Therefore, developing effective methods for separating sphalerite and pyrite is a key step in improving the comprehensive utilization of zinc resources.

Foam flotation is currently the main industrial method for separating sulfide minerals [5,6,7]. It utilizes the difference in hydrophobicity of mineral surfaces: bubbles selectively adsorb onto hydrophobic particles, carrying them into the foam layer, while hydrophilic minerals remain in the pulp, thus achieving separation [8,9,10,11,12,13]. Among various collectors, xanthate is widely used due to its low cost and strong affinity for sulfide mineral surfaces [4,14,15]. However, the flotation separation of sphalerite and pyrite remains challenging due to their similar natural flotation properties and the poor selectivity of traditional xanthates for both [1,16,17]. In practical applications, activators (such as copper salts) are often used to improve the flotation recovery of sphalerite, but these activators often simultaneously activate pyrite, further complicating the separation process [16,18,19]. Therefore, the development and application of selective inhibitors to suppress pyrite flotation has become crucial.

Pyrite inhibitors are mainly classified into three categories: inorganic, organic, and microbial [3,20,21]. Currently, most organic and microbial inhibitors are still in the laboratory research stage, limited by cost and environmental factors [1,3,20]. In industrial practice, the widespread use of lime has caused many operational and environmental problems, such as pipe scaling and high carbon emissions generated during production, which contradicts the “dual carbon” goal [1,6,22]. Other inorganic inhibitors also face serious challenges, such as the high toxicity of cyanide, which poses a threat to health, ecosystems, and precious metal recovery [9,23,24], while oxidants (such as H_2_O_2_ and KMnO_4_) usually require strict process control and have insufficient selectivity [3,25]. Given these limitations, the development of efficient and environmentally friendly pyrite inhibition technologies is crucial for both economic development and environmental protection [26,27].

Previous studies have shown that novel reagent systems, such as FeCl_3_-CuSO_4_-APTT/BX, can improve sphalerite–pyrite separation [16]. However, their effectiveness is strongly dependent on highly acidic conditions (pH 3~4), raising concerns about equipment corrosion, increased maintenance costs, and safety risks, thus hindering their industrial application. To understand this pH dependence and explore alternatives, we performed solution chemistry analysis. The results showed that the selective inhibition of FeCl_3_ is achieved by the formation of Fe(OH)_2_^+^ cation clusters within a narrow pH window of 3.0~4.0. Importantly, further calculations showed that the environmentally friendly FeSO_4_-NaH_2_PO_4_ system can generate similar active species FeH_2_PO_4_^+^, even under near-neutral conditions (pH ≈ 6.0), providing a potentially more sustainable and practical inhibition pathway.

Based on the above analysis, this study employs a novel flotation system, FeSO_4_+NaH_2_PO_4_-CuSO_4_-SIBX, for the flotation separation of sphalerite and pyrite. Key parameters such as reagent dosage and pulp pH were optimized through single-mineral and artificially mixed mineral flotation experiments. Analytical techniques, including contact angle, zeta potential, adsorption capacity measurement, hydrophobic aggregation, and X-ray photoelectron spectroscopy (XPS), were used to further elucidate the selective separation mechanism of sphalerite and pyrite.

## 2. Results

### 2.1. Micro-Flotation Experiment Results

#### 2.1.1. Single Mineral Flotation

**Figure 1:**
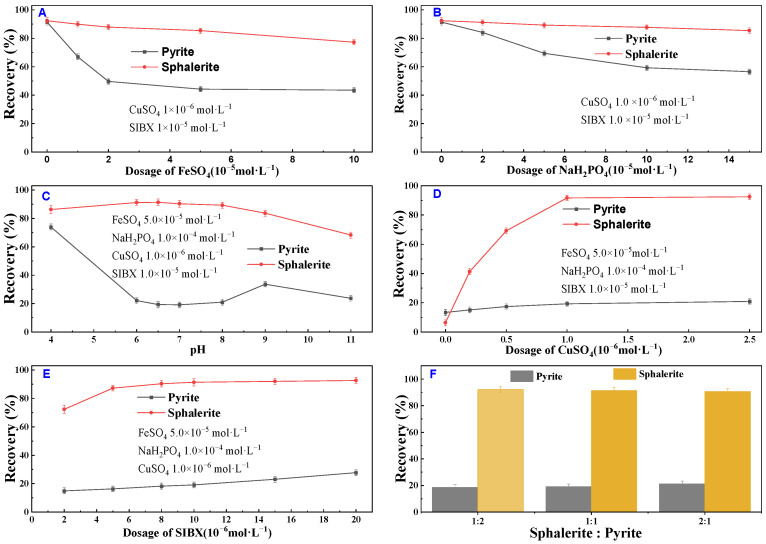
Micro-flotation results of single minerals (**A**–**C**) and mixed minerals (**D**–**F**): (**A**) Effect of FeSO_4_ dosage on flotation recovery when FeSO_4_ is used alone as an inhibitor. (**B**) Effect of NaH_2_PO_4_ dosage on flotation recovery when NaH_2_PO_4_ is used alone as an inhibitor. (**C**) Effect of pH on the flotation recovery of the FeSO_4_-NaH_2_PO_4_ composite inhibitor system. (**D**) Effect of CuSO_4_ dosage on the flotation recovery of the FeSO_4_-NaH_2_PO_4_ combined inhibitor system. (**E**) Effect of isobutyl xanthate (SIBX) dosage on the flotation recovery of the FeSO_4_-NaH_2_PO_4_ combined inhibitor system. (**F**) Effect of the proportion of mixed minerals on the flotation recovery of the FeSO_4_-NaH_2_PO_4_ composite inhibitor system.

The single-mineral flotation results are shown in Figure 1A–C. Figure 1A shows the effect of FeSO_4_ dosage on mineral flotation recovery in the presence of 1 × 10^−6^ mol·L^−1^ CuSO_4_ and 1 × 10^−5^ mol·L^−1^ SIBX. As the FeSO_4_ concentration increased from 0 to 2 × 10^−5^ mol·L^−1^, the pyrite flotation recovery dropped sharply from 91.4% to 49.7% (a decrease of 41.7%). In contrast, the sphalerite flotation recovery only decreased slightly, from 92.4% to 87.9% (a decrease of 4.5%). When the FeSO_4_ concentration was 5 × 10^−5^ mol·L^−1^, the pyrite flotation recovery stabilized at ~44%, while the sphalerite flotation recovery remained around 85%, with the maximum difference in flotation recovery being approximately 41%. These results confirm the highly selective inhibitory effect of FeSO_4_ on pyrite relative to sphalerite, suggesting the feasibility of separating these two minerals based on this mechanism.

Figure 1B shows the flotation response of pyrite and sphalerite using NaH_2_PO_4_ alone. As the NaH_2_PO_4_ concentration increased from 0 to 2 × 10^−5^ mol·L^−1^, the pyrite flotation recovery gradually decreased from 91.4% to 84.1%. In contrast, the sphalerite flotation recovery was almost unaffected, decreasing by only about 1%. This indicates that the inhibition effect of NaH_2_PO_4_ alone is limited within this concentration range. However, when the NaH_2_PO_4_ concentration exceeded 1 × 10^−4^ mol·L^−1^, the decrease in pyrite flotation recovery was more significant, dropping from 84.1% to 59.3% (a further decrease of 24.8%). This accelerated inhibition at high concentrations may be due to the competitive adsorption of H_2_PO_4_^−^ anions on the pyrite surface by active sites, thus interfering with collector adhesion. The key point is that even when used alone, NaH_2_PO_4_ is not very effective, but it can still selectively interact with the pyrite surface, which suggests that it may have a synergistic effect with FeSO_4_, possibly by enhancing or stabilizing the hydrophilic layer formed by iron species.

To evaluate the synergistic effect, a composite inhibitor system consisting of 5 × 10^−5^ mol·L^−1^ FeSO_4_ and 1 × 10^−4^ mol·L-1 NaH_2_PO_4_ was established based on previous single-factor experiments. Subsequently, flotation experiments were conducted at different pH values under the condition of fixed concentrations of CuSO_4_ 1 × 10^−6^ mol·L^−1^ and SIBX 1 × 10^−5^ mol·L^−1^. As shown in Figure 1C, the pH value has a significant impact on separation efficiency. The flotation recovery of pyrite showed a complex trend, first decreasing and then slightly increasing, before decreasing again at pH values above 9. In contrast, the flotation recovery of sphalerite first increased and then gradually decreased with increasing pH value. Notably, excellent selectivity was obtained in the near-neutral pH range (6~8). The flotation recovery of pyrite decreased significantly (19.1%~22.1%), while the flotation recovery of sphalerite remained at a high level (89.3%~91.4%), resulting in a consistently large difference in flotation recovery exceeding 68.3%. This indicates that efficient separation is feasible under environmentally friendly and near-neutral conditions.

The performance represents a significant advancement over the previously reported FeCl_3_ system, which requires highly acidic conditions (pH 3~4) [16]. Operating under near-neutral pH conditions reduces the risk of equipment corrosion and harmful H_2_S generation. The underlying mechanism (supported by solution chemistry analysis) explains this optimal pH range: at pH 6–8, the dominant active species FeH_2_PO_4_^+^ readily forms stable ferrous phosphate complexes on the pyrite surface, achieving selective inhibition. However, in strongly alkaline environments (pH > 10), Fe^2+^ precipitates as Fe(OH)_2_ (>60%), weakening its synergistic effect with phosphate. Simultaneously, high concentrations of OH^−^ compete with SIBX for adsorption sites on sphalerite, reducing its flotation recovery.

#### 2.1.2. Mixed Minerals Flotation

To verify and optimize the application of the FeSO_4_-NaH_2_PO_4_ composite inhibitor system in practical separation, we systematically studied the effects of key parameters, such as the dosage of activator CuSO_4_ and collector SIBX, and the ore mixing ratio, on the flotation performance of sphalerite–pyrite mixtures.

The effect of CuSO_4_ dosage on mineral flotation recovery is shown in Figure 1D. The tests were conducted at a natural pH of 6.0–8.0, with 5 × 10^−5^ mol·L^−1^ FeSO_4_, 1 × 10^−4^ mol·L^−1^ NaH_2_PO_4_, and 1 × 10^−5^ mol·L^−1^ SIBX conditions. CuSO_4_ showed a high selective activation effect on sphalerite. As the amount of CuSO_4_ increased to 2.5 × 10^−6^ mol·L^−1^, the recovery of sphalerite increased from 6.4% to 92.5%, while the recovery of pyrite remained at a low level of about 20.9%. This selectivity is due to the easy ion exchange reaction of Cu^2+^ on the surface of sphalerite (ZnS + Cu^2+^ →CuS + Zn^2+^), generating Cu-S sites with high affinity for xanthate [5]. Conversely, the pre-formed hydrophilic layer on the pyrite surface (composed of Fe^2+^/H_2_PO_4_^−^ complex) significantly inhibits Cu^2+^ adsorption. The optimal CuSO_4_ dosage is considered to be 1 × 10^−6^ mol·L^−1^, at which the flotation recovery of sphalerite reaches 91.7%, while the flotation recovery of pyrite is only 19.1%, a difference of 72.6%.

The effect of SIBX concentration was investigated at the optimal CuSO_4_ dosage of 1 × 10^−6^ mol·L^−1^, as shown in Figure 1E. With the SIBX concentration increased from 2 × 10^−6^ mol·L^−1^ to 1 × 10^−5^ mol·L^−1^, the flotation recovery of sphalerite increased from 72.4% to 91.7% and then stabilized. Notably, the flotation recovery of pyrite remained below 19.1% at all tested concentrations, demonstrating effective depression of pyrite. This outcome reveals a broad window for selective separation. The slight increase in pyrite flotation recovery at extremely high SIBX concentrations is attributed to the non-selective adsorption of excess xanthate. Therefore, 1 × 10^−5^ mol·L^−1^ was chosen as the optimal SIBX dosage.

With optimized reagent formulations, the performance of the system was tested at different sphalerite to pyrite mass ratios (see Figure 1F). The separation effect remained significant: sphalerite recovery consistently exceeded 90.7%, while pyrite recovery was suppressed to below 21.1%, maintaining stable separation efficiency. This robustness indicates that the composite inhibitor system is insensitive to small fluctuations in feed composition, a key characteristic for its potential industrial applications.

### 2.2. Mineral Surface Hydrophobicity Analysis

To elucidate the mechanism behind the observed flotation separation selectivity, we investigated the changes in surface hydrophobicity induced by the reagent scheme using mineral agglomeration experiments and contact angle measurements.

**Figure 2:**
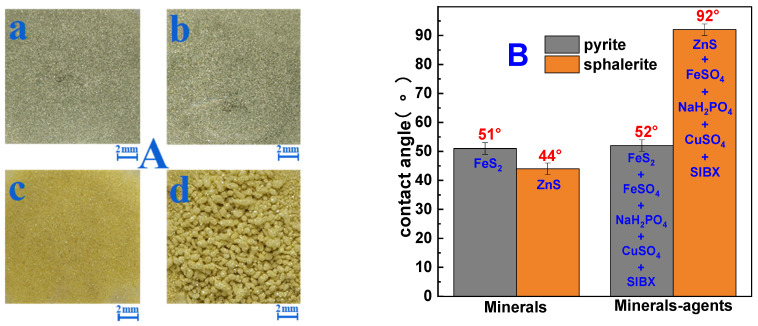
The optical microscopy (**A**) (**a**: pyrite, **b**: pyrite after treated, **c**: sphalerite, **d**: sphalerite after treated) and contact angle (**B**) of minerals before and after interaction with reagents.

The hydrophobic aggregation of minerals provides direct visual evidence, which is shown in Figure 2A. No obvious aggregation was observed in untreated pyrite (Figure 2A(a)) and sphalerite (Figure 2A(c)). After treatment with the novel flotation system FeSO_4_+NaH_2_PO_4_-CuSO_4_-SIBX, pyrite particles (Figure 2A(b)) remained dispersed, indicating that their hydrophilicity was maintained; however, sphalerite particles (Figure 2A(d)) formed larger aggregates (300~1000 μm), confirming a significant enhancement in their surface hydrophobicity.

The contact angle measurements further quantitatively confirmed this comparison (see Figure 2B). The initial contact angles of sphalerite (44°) and pyrite (51°) were similar. After reagent treatment, the contact angle of sphalerite increased sharply to 92°, indicating a highly hydrophobic surface. Conversely, the contact angle of pyrite increased only slightly to 52°, remaining within the hydrophilic range.

### 2.3. Zeta Potential Test

**Figure 3:**
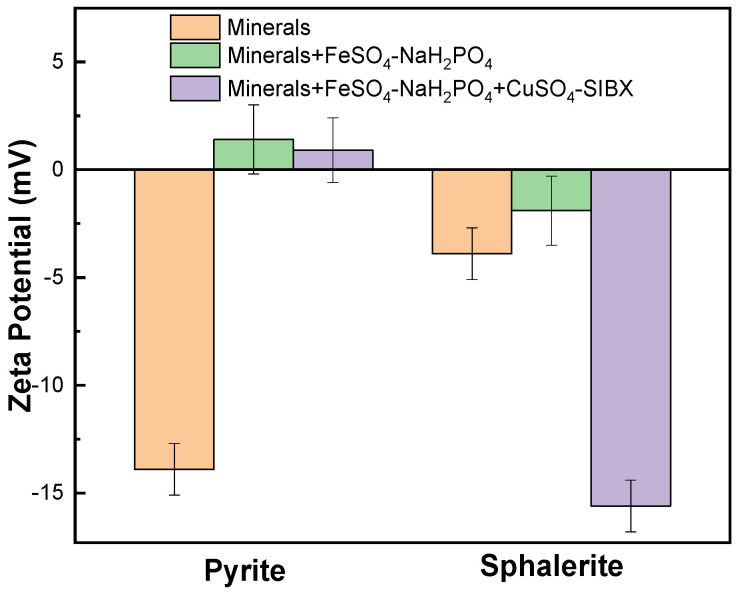
Zeta potential of minerals before and after interaction with reagents under natural pH conditions (6.0~8.0).

Zeta potential measurements provide direct evidence for the selective separation mechanism (Figure 3). The zeta potential of sphalerite and pyrite is −3.9 mV and −13.9 mV, respectively. Significant differences emerged after treatment with the FeSO_4_-NaH_2_PO_4_ composite inhibitor. The zeta potential of pyrite shifted significantly from −13.9 mV to +1.4 mV (Δ = +15.3 mV). This significant positive shift clearly indicates that positively charged substances selectively adsorbed onto the pyrite surface. In contrast, the potential of sphalerite changed only slightly to −1.9 mV (Δ = +2.0 mV), indicating minimal interaction with the inhibitor. Subsequent addition of CuSO_4_ and SiBX further altered the surface properties. The zeta potential of sphalerite became more negative (−15.6 mV, Δ = −13.7 mV), confirming the successful chemisorption of the anionic collector SIBX on its activated surface. Meanwhile, the potential of pyrite remained almost unchanged at +0.9 mV. This stability indicates that the pre-adsorbed hydrophilic layer effectively prevented SIBX anions from approaching the pyrite surface. The strong selective adsorption of the reagent on each mineral quantitatively explains the flotation separation selectivity: pyrite was selectively hydrophilized and shielded from collector adhesion, while sphalerite was effectively activated and hydrophobic.

### 2.4. Adsorption Amount of Reagent on Mineral Surface

**Figure 4:**
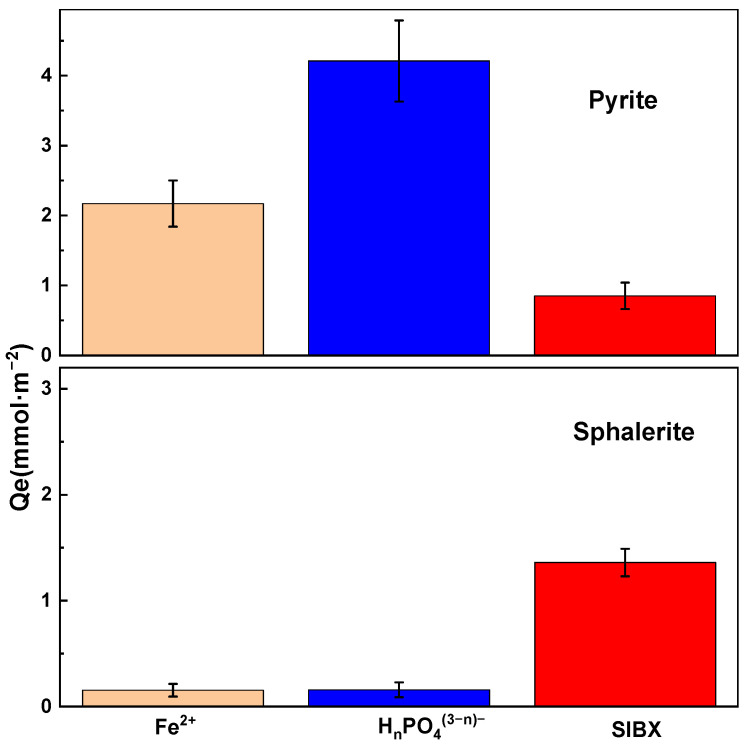
Adsorption amount of reagents on the mineral surface.

Quantitative adsorption measurements provide conclusive evidence for the proposed selective interactions (see Figure 4). The adsorption amount of the inhibitor components on pyrite (Fe^2+^ 2.17 mmol·m^−2^, H_2_PO_4_^−^ 4.21 mmol·m^−2^) is an order of magnitude higher than that on sphalerite (Fe^2+^ 0.15 mmol·m^−2^, H_2_PO_4_^−^ 0.16 mmol·m^−2^). This significant contrast confirms the strong preferential affinity of the FeSO_4_-NaH_2_PO_4_ system for the pyrite surface. Conversely, the adsorption amount of the collector SIBX on pyrite (0.85 mmol·m^−2^) is significantly lower than that on sphalerite (1.36 mmol·m^−2^). This inverse relationship stems directly from previous surface modifications: the abundant hydrophilic layer on pyrite effectively hinders collector adsorption, while the relatively clean and Cu^2+^-activated sphalerite surface readily chemisorbs xanthate. These quantitative adsorption data integrate previous findings: The abundant uptake of hydrophilic substances by pyrite, coupled with its limited collector adsorption, explains its persistent hydrophilicity. Simultaneously, the extremely low uptake of inhibitors by sphalerite, coupled with its high collector loading, explains its strong hydrophobicity. Therefore, the adsorption results provide robust quantitative validation of the selective inhibition and activation mechanisms driving efficient flotation separation.

### 2.5. XPS Analysis

**Figure 5:**
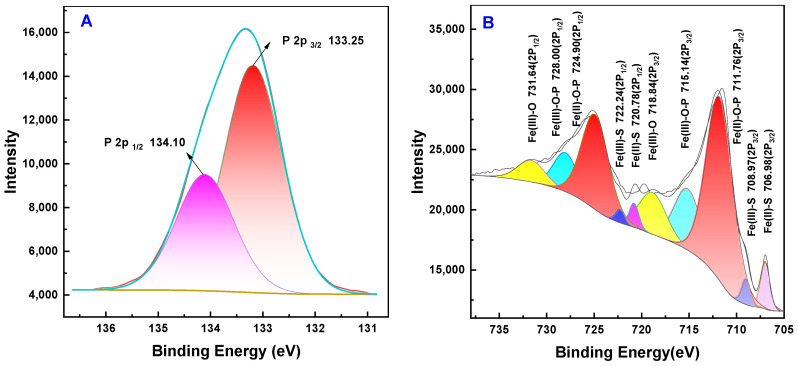
The high-resolution XPS spectra of P 2p (**A**) and Fe 2p (**B**) on the pyrite surface treated by FeSO_4_-NaH_2_PO_4._

The high-resolution XPS spectra of P 2p and Fe 2p on the pyrite surface treated with FeSO_4_-NaH_2_PO_4_ are listed in Figure 5, and their XPS peak assignments are displayed in Table 1. The results in Figure 5 and Table 1 show that, after FeSO_4_-NaH_2_PO_4_ treatment, the P 2p XPS band at around 133.37 and 134.10 eV appeared on the pyrite surface, which were assigned to the P-O bond in phosphate [28,29,30]. The Fe 2p_3/2_ peak can be fitted into five components with binding energies of 706.98 eV, 708.97 eV, 711.76 eV, 715.14 eV, and 718.84 eV, corresponding to Fe(II)-S, Fe(III)-S, Fe(II)-O-P, Fe(III)-O-P, and Fe(III)-O species, respectively [3,31,32,33,34], suggesting that at the molecular level the composite inhibitor forms a hydrophilic Fe(II)/Fe(III)–phosphate complex on the pyrite surface.

### 2.6. Solution Chemical Analysis

**Figure 6:**
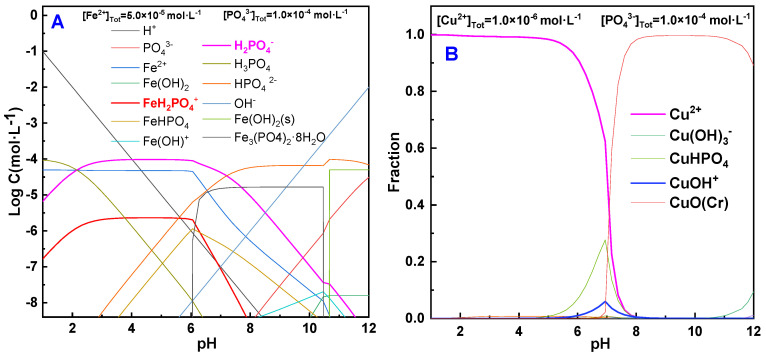
Distribution diagrams of different components in Fe^2+^-PO_4_^3−^ solutions (**A**) and Cu^2+^-PO_4_^3−^ solutions (**B**) as a function of pH.

The solution chemistry of the Fe^2+^-PO_4_^3−^ and Cu^2+^-PO_4_^3−^ systems was investigated using MEDUSA (Vers. 18 Feb. 2004) [35]. The relevant results are presented in Figure 6. Figure 6A displays the Fe^2+^-PO_4_^3−^ concentration–pH curves. When FeSO_4_ and NaH_2_PO_4_ were added, the natural pH of the slurry was approximately 6.0, and the dominant species were Fe^2+^, FeH_2_PO_4_^+^, and H_2_PO_4_^−^. Subsequently, after the addition of SIBX, within the optimal flotation separation pH range of 6.0~8.0, the thermodynamically stable solid Fe_3_(PO_4_)_2_·8H_2_O became the dominant species. XPS detected surface Fe-O-P complexes, consistent with the precipitation or surface condensation of phosphate-rich phases, forming a hydrophilic layer on the pyrite surface [5].

Figure 6B shows the Cu^2+^-PO_4_^3−^ concentration–pH curves. At pH 6.0~8.0, the active components of Cu(II) are mainly Cu^2+^ and CuOH^+^, both of which are positively charged cations or cationic groups. Although CuHPO_4_ has a proportion of 0.3 at pH 7, it is electrically neutral.

## 3. Discussion

**Figure 7:**
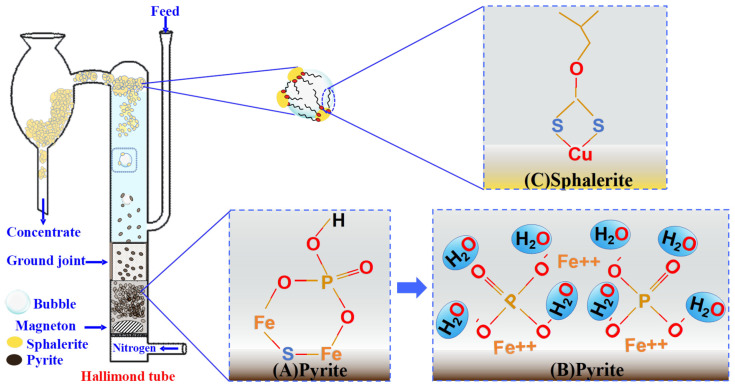
Possible mechanisms for the flotation separation of sphalerite and pyrite.

Based on solution chemistry and surface analysis methods, such as contact angle, aggregation experiment, zeta potential, and XPS, a molecular-level mechanism for the selective inhibition of pyrite by the FeSO_4_+NaH_2_PO_4_-CuSO_4_-SIBX system under near-neutral conditions was proposed. The potential flotation separation mechanism is shown in Figure 7.

The addition of FeSO_4_ and NaH_2_PO_4_ to the slurry generates the key cationic species FeH_2_PO_4_^+^. Pyrite, with its extremely high negative zeta potential (−13.9 mV), strongly attracts FeH_2_PO_4_^+^, while sphalerite, with its lower zeta potential (−3.9 mV), exhibits a much weaker affinity. This electrostatic attraction advantage drives the selective adsorption of FeH_2_PO_4_^+^ onto pyrite [36,37]. Subsequent absorption stabilizes the complex on the pyrite surface. The Fe(II) centers can coordinate with surface S atoms, while O atoms bind to surface Fe sites, potentially forming stable six-membered rings, thus firmly anchoring the inhibitor [38,39].

Upon addition of SIBX, the pH value changed to 6.0~8.0. Within this range, the adsorbed layer transformed into a more stable hydrophilic substance, particularly Fe_3_(PO_4_)_2_·8H_2_O, see reactions (1)–(2), which dominated the surface and effectively passivated pyrite, preventing collector adsorption [40,41,42].

FeH_2_PO_4_^+^ + OH^−^ = FeHPO_4_ + H_2_O (1)

2FeHPO_4_ + Fe^2+^ + 2OH^−^ + 6H_2_O = Fe_3_(PO_4_)_2_·8H_2_O (2)

Meanwhile, Cu^2+^ activates sphalerite through ion exchange (ZnS + Cu^2+^ → CuS + Zn^2+^) [43]. The generated Cu-S sites can adsorb SIBX through strong chemical interaction, enhancing the hydrophobicity of zinc sphalerite [19,21,44].

Figure 1C clearly shows the key role of pH value, which can be explained by the distribution of species in the solution. When the pH value is below 4, H_3_PO_4_ dominates and has a weak complexing ability with Fe^2+^, the pyrite surface has less negative charge, resulting in a poor inhibition effect. At pH 6~8, FeH_2_PO_4_^+^ forms and adsorbs to the optimal state, thereby achieving effective inhibition [5]. Above pH 8, FeH_2_PO_4_^+^ converts to FeHPO_4_, losing its positive charge and electrostatic advantage. Above pH 10, surface Fe(II) precipitates as hydrophilic Fe(OH)_2_, further reducing pyrite recovery [45].

The contrast in surface charge after inhibitor treatment also enables selective activation: the positively charged pyrite surface repels cations Cu^2+^/CuOH^+^, while the negatively charged zincblende surface attracts them, thereby promoting ion exchange and subsequent SIBX chemisorption [46].

In summary, the selective adsorption, chemisorption, and precipitation of electrostatically driven hydrophilic iron phosphate on pyrite, along with the simultaneous hydrophobication of copper-activated sphalerite, collectively explain the efficient flotation separation under near-neutral pH conditions. While this model is consistent with our experimental observations, further investigation is needed to elucidate more details of the interfacial interactions.

## 4. Materials and Methods

### 4.1. Materials

Sphalerite (ZnS) and pyrite (FeS_2_) mineral samples were obtained from Guangzhou Ye’s Mineral Specimens Co., Ltd. (Guangzhou, China). For micro-flotation experiments, bulk mineral samples were crushed, manually sorted, ground, and dry-sieved to obtain particles with a diameter of 38–75 μm. Particles smaller than 38 μm were further ground to below 5 μm for zeta potential and XPS measurements. All processed samples were stored in sealed glass vials and refrigerated to minimize surface oxidation.

**Figure 8:**
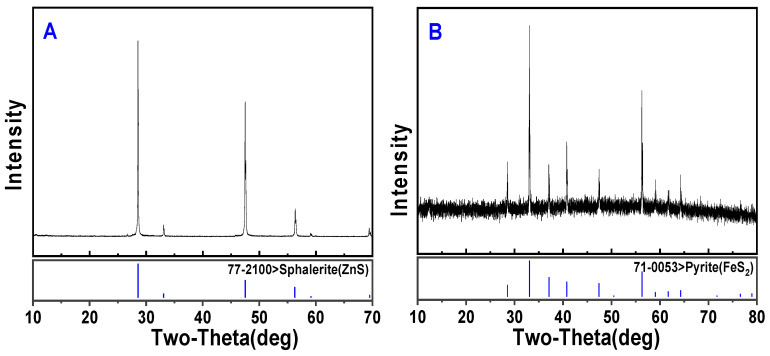
XRD of sphalerite (**A**) and pyrite (**B**).

The purity of the minerals was characterized using X-ray diffraction (XRD; D8 ADVANCE, Bruker GmbH, Mannheim, Germany) and X-ray fluorescence spectrometry (XRF; S4 PIONEER, Bruker GmbH, Germany). XRD was operated with Cu Kα radiation under 40 kV and 40 mA, scanning from 5° to 80° (2θ) at 2°/min. For XRF, samples were dried, pressed into pellets (4 g + 0.5 g H_3_BO_3_), and then analyzed at 30 kV and 100 mA. The purity of both minerals exceeded 97%, with quartz identified as the major impurity (see Figure 8 and Table 2). Chemical analysis showed that sphalerite contained 65.51% Zn, 32.38% S, and 0.44% Fe, while pyrite contained 45.53% Fe and 52.34% S. The contents of other impurity elements (e.g., Pb, Si, Ca) were all less than 1% of the total composition.

The following analytical grade reagents were used in this experiment: ferrous sulfate heptahydrate (FeSO_4_·7H_2_O) and sodium dihydrogen phosphate (NaH_2_PO_4_) as a composite depressant; copper sulfate (CuSO_4_) as an activator; hydrochloric acid (HCl) and sodium hydroxide (NaOH) for pH adjustment; and methyl isobutyl carbinol (MIBC) as a frother. All the above reagents were purchased from Aladdin Industrial Co., Ltd. (Shanghai, China). Industrial-grade isobutyl xanthate (SIBX, purity ≥ 95%) served as the collector, which was obtained commercially. All solutions were prepared using distilled water.

### 4.2. Micro-Flotation Tests

**Figure 9:**
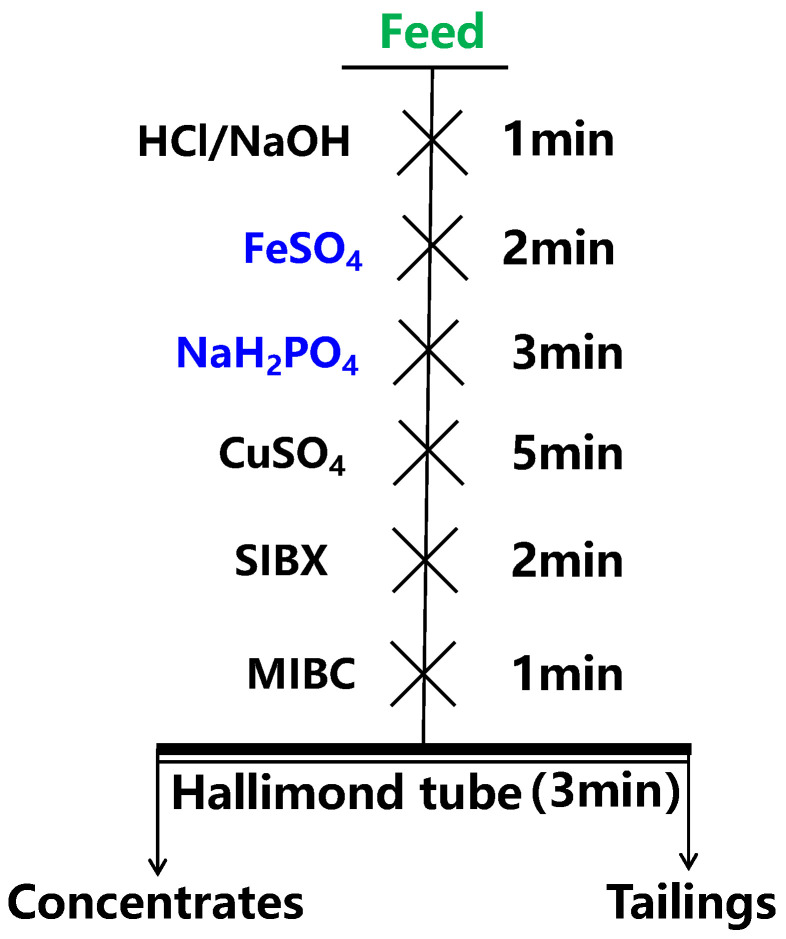
The flowsheet of micro-flotation experiments.

Micro-flotation experiments were conducted in a modified Hallimond tube, and the flotation process flow diagram is shown in Figure 9. For each experiment, 2.0 g of mineral sample was weighed, placed in a beaker, and ultrasonically cleaned three times (1 min each time) with water to remove the oxide layer on the mineral surface [47]. The pH was adjusted to the desired value using dilute hydrochloric acid or sodium hydroxide solution, and the mixture was allowed to stand for 1 min. Subsequently, under stirring, the inhibitors FeSO_4_ and NaH_2_PO_4_, the activator CuSO_4_, the collector SIBX, and the frother MIBC were added sequentially to reach the preset concentration. The total volume of the suspension was controlled to be 220 mL by adding water, and the MIBC concentration was 1.5 × 10^−4^ mol·L^−1^. The pH value of the mixture was measured again before flotation. The slurry was transferred to a Hallimond tube, the nitrogen flow rate was adjusted to 0.2 L·min^−1^, and the flotation time was 3 min. The foam products (concentrate) and the products in the tank (tailings) were collected separately. The concentrate and tailings were filtered, dried, and weighed, and the flotation recovery was calculated. The recovery of pure minerals was calculated according to Equation (3). For mixed minerals, the recovery rates of sphalerite and pyrite were calculated according to Equations (4) and (5), respectively [3,16]. All experimental results were the average values of three parallel experiments.

(3) $$\varepsilon =\frac{m_{1}}{m_{1}+m_{2}}\times 100\%$$

(4) $$\varepsilon_{\mathrm{Sphalerite}}=\frac{\beta_{1}\times m_{1}}{\beta_{1}\times m_{1}+\beta_{2}\times m_{2}}$$

(5) $$\varepsilon_{\mathrm{Pyrite}}=\frac{\left(1-\beta_{1}\right)\times m_{1}}{\left(1-\beta_{1}\right)\times m_{1}+\left(1-\beta_{2}\right)\times m_{2}}$$

In the above equations, *ε* represents the flotation recovery (w%), *β* denotes the grade of sphalerite (w%), which was calculated from Zn content determined by titration, and m signifies the weight (g). Subscripts 1 and 2 stand for concentrates and tailings, respectively.

### 4.3. Surface Hydrophobicity Characterization

The hydrophobicity of mineral surfaces was assessed through contact angle measurements and direct observation of particle agglomeration.

Contact angle measurements were performed using a JC2000C goniometer (Shanghai Zhongchen, Shanghai, China). Mineral flakes were sequentially polished with SiC sandpaper of different grits and finally polished with a 20 nm Al_2_O_3_ suspension. After rinsing with distilled water and drying under a nitrogen stream, the samples were immediately used for testing. Contact angles were measured at three different locations on each sample surface, including the untreated (fresh) surface and the surface treated sequentially with FeSO_4_, NaH_2_PO_4_, CuSO_4_, and SIBX (simulating the flotation process treatment scheme) [11,15]. The reported values are the average of the three measurements.

Particle agglomeration experiments were conducted to visually assess hydrophobicity under simulated flotation conditions. Using an NP-800TRF optical microscope (Jiangnan Digital, Wuxi, China), we observed the aggregation state of mineral particles in the presence of different reagent systems [16]. The sample preparation (e.g., reagent addition order and conditioning times) strictly followed the micro-flotation test procedure, except that no frother (MIBC) was added and no nitrogen was introduced.

### 4.4. Zeta Potential Measurement

The zeta potential of the mineral particles was measured with a ZetaPlus analyzer (Brookhaven, Nashua, NH, USA). For each test, 50 mg of finely ground sample (<5 μm) was dispersed in 50 mL of a 1 × 10^−3^ mol·L^−1^ KCl background electrolyte solution. Fresh grinding was performed prior to measurement to reduce surface oxidation. After adjusting the suspension to the target pH, it was conditioned sequentially with the depressant (FeSO_4_-NaH_2_PO_4_) and the activator/collector system (CuSO_4_-SIBX). Each measurement was repeated 10 times, and the reported values represent the average values [48,49].

### 4.5. XPS Detection

Surface chemical states of pyrite treated with the depressant system were analyzed by XPS using an ESCALAB 250Xi spectrometer (Thermo Scientific, Waltham, MA, USA). The pyrite samples were treated with the FeSO_4_-NaH_2_PO_4_ depressant under natural pH (6.0–8.0), adopting the same treatment process as the flotation tests. Measurements were performed with a monochromated Al Kα X-ray source (hv = 1486.71 eV) under an ultra-high vacuum better than 10^−8^ Torr [50]. High-resolution spectra were acquired with a dwell time of 300 ms per data point and were averaged over 16 scans to enhance the signal-to-noise ratio. All spectra were charge-corrected by referencing the adventitious carbon C 1s peak to 284.80 eV. Peak fitting and subsequent chemical state analysis were conducted using the Thermo Avantage software package 5.52.

### 4.6. Adsorption Experiment

Briefly, 1.0 g of mineral sample was sequentially treated with FeSO_4_, NaH_2_PO_4_, CuSO_4_, and SIBX to simulate the flotation procedure. After each reagent treatment, the suspension was kept standing for 1 min. Subsequently, 10 mL of filtrate was collected using a syringe filter. The residual Fe(II) concentration in the filtrate was determined via the o-phenanthroline spectrophotometric method. The residual phosphorus content was measured by the molybdenum blue spectrophotometric method. The concentration of residual SIBX was detected using UV spectrophotometry at a wavelength of 300 nm.

The adsorption amount was calculated according to the difference between its initial and residual concentration, which is shown in Equation (6).

(6) $$Q=\frac{\left(C_{0}-C\right)\times V}{M\times S}$$

Here, Q is the adsorption quantity (mol·m^−2^); V is the solution volume (L); C_0_ and C are the reagents’ initial and residual concentration (mol·L^−1^); and M and S are the mass (g) and specific surface area (m^2^·g^−1^) of pyrite or sphalerite, respectively.

## 5. Conclusions

The FeSO_4_-NaH_2_PO_4_-CuSO_4_-SIBX flotation system can efficiently separate sphalerite and pyrite. The separation efficiency of the mixed minerals was consistently higher than 4.3, indicating that the system has good adaptability to different mineral compositions.

Mechanistically, Fe^2+^ from FeSO_4_ and H_2_PO_4_^−^ from NaH_2_PO_4_ synergistically adsorb onto the pyrite surface, forming hydrophilic iron phosphate complexes, such as FeH_2_PO_4_^+^ and Fe_3_(PO_4_)_2_·8H_2_O. This complex layer enhances the hydrophilicity of pyrite and hinders its subsequent interactions with Cu^2+^ and SIBX. Simultaneously, CuSO_4_ selectively activates sphalerite via Cu^2+^ exchange reactions, while SIBX further hydrophobizes the surface by forming stable S-Cu bonds with adsorbed copper. The synergistic effect of these four reagents significantly amplifies the flotation differences between the two minerals.

Compared to traditional strongly alkaline or weakly acidic systems, this system operates under near-neutral to weakly alkaline conditions, thus mitigating equipment corrosion. Furthermore, the reagents used in the study are inexpensive and have low toxicity, aligning with environmental sustainability goals. Therefore, this work provides a new theoretical insight and a practical technical strategy for the green separation of zinc sulfide ores.

In summary, the developed system yields satisfactory results for single minerals and artificially mixed samples. To explore its engineering potential, follow-up research will focus on performance evaluation using real polymetallic ores.

## Figures and Tables

**Table 1:** The fitting and assignment of P 2p and Fe 2p XPS.

Components	Binding Energy/eV	FWHM eV	Atomic %
C 1s	284.80	1.51	44.61
P 2p	133.37	1.78	23.69
S 2p	162.46	0.92	9.77
Fe 2p	711.77	3.97	21.93

**Table 2:** Chemical composition of sphalerite and pyrite samples.

	Elemental Analysis Results (%)										
**Elements**	Zn	S	Fe	Pb	O	Si	Ca	Mn	Al	Mg	Others
**Sphalerite**	65.51 *	32.38 *	0.44	0.01	1.06	0.24	0.06	0.02	0.11	0.05	0.12
**Pyrite**	0.06	52.34 *	45.53 *	0.01	1.28	0.31	0.11	-	0.16	0.09	0.11

* The contents of Zn in sphalerite and Fe in pyrite were determined by titration, while sulfur in both minerals was measured using the gravimetric method, and the contents of other components are the results of XRF.

## Data Availability

The original contributions presented in this study are included in the article. Further inquiries can be directed to the corresponding authors.
